# “Family Burnout” of psychiatric patients: its role during the COVID-19 pandemic

**DOI:** 10.1192/j.eurpsy.2022.499

**Published:** 2022-09-01

**Authors:** F. Franza, A. Vacca, M.V. Minò, B. Solomita, F. Papa, A. De Paola, A. Franza

**Affiliations:** 1 Psychiatric Rehabilitation Centre Villa dei Pini, Psychiatry, Avellino, Italy; 2 ASL Taranto, Mental Health Department, Taranto, Italy; 3 Psychiatric Rehabilitation Centre Don Tonino Bello, Assoc Mitag, Brindisi, Italy; 4 Neamente Association, Neuroscience, Mercogliano (AV), Italy

**Keywords:** Burnout caregivers, Family Members, Covid-19, Stress

## Abstract

**Introduction:**

Family members caregivers (FMCs) of patients with severe psychiatric disorders (SMPD) are subjected to a complex system of fatigue and stress. FMCs can be subjected to a care burden defined as “Family Burnout”. Caring of family members of patients affected by psychiatric disorder suffered an additional burden during the pandemic period.

**Objectives:**

To investigate the stress, burnout and compassion fatigue in FMCs during the pandemic vs non-pandemic period.

**Methods:**

In our observational study we recruited family members (FMCs) of SMPDs (DSM-5). The severity was assessed with BPRS > 31; from March 2021 to July 2021 (T1), in 66 FMCs (38 females, 28 men) that completed following questionnaires: CBI (Caregiver Burden Inventory), ProQOL (compassion satisfaction and compassion fatigue (burnout and secondary trauma) subscales]. These data (T1) were compared with the scores obtained in the same family members in 2019 (T0) in a pre-pandemic period.

**Results:**

ProQOL data /T1) have a higher total score than those observed in a previous study (T0). They show a lower main score in Compassion Satisfaction (CS) subscale [T1 vs T0; 34.27 vs 38.89 (p < .00.5). CS subscale T0 vs T1= 34.84% vs 12.12%). High levels of burnout were found in 28.79% (T1) vs 13.64% (T0) of FMCs group. Similar results showed in the Secondary Trauma subscale and CBI with higher scores in T1 vs T0.

**Conclusions:**

The comparative mean results (2019 vs 2021) showed that in the same group of FMCs, the mean values obtained with same scales were higher during the lockdown. During health crisis, FMCs of psychiatric patients are subjected to high levels of stress.

**Disclosure:**

No significant relationships.

